# Intratumoral and peritumoral radiomics predict pathological response after neoadjuvant chemotherapy against advanced gastric cancer

**DOI:** 10.1186/s13244-023-01584-6

**Published:** 2024-01-25

**Authors:** Chenchen Liu, Liming Li, Xingzhi Chen, Chencui Huang, Rui Wang, Yiyang Liu, Jianbo Gao

**Affiliations:** 1 Department of Radiology, The First Affiliated Hospital of Zhengzhou University, No. 1, East Jianshe Road, Zhengzhou, 450052 Henan China; 2 Department of Research Collaboration, R&D Center, Beijing Deepwise & League of PHD Technology Co., Ltd, Beijing, China

**Keywords:** Gastric cancer, Peritumoral radiomics, Neoadjuvant chemotherapy, X-ray computed tomography, Pathological response

## Abstract

**Background:**

To investigate whether intratumoral and peritumoral radiomics may predict pathological responses after neoadjuvant chemotherapy against advanced gastric cancer.

**Methods:**

Clinical, pathological, and CT data from 231 patients with advanced gastric cancer who underwent neoadjuvant chemotherapy at our hospital between July 2014 and February 2022 were retrospectively collected. Patients were randomly divided into a training group (*n* = 161) and a validation group (*n* = 70). The support vector machine classifier was used to establish radiomics models. A clinical model was established based on the selected clinical indicators. Finally, the radiomics and clinical models were combined to generate a radiomics–clinical model. ROC analyses were used to evaluate the prediction efficiency for each model. Calibration curves and decision curves were used to evaluate the optimal model.

**Results:**

A total of 91 cases were recorded with good response and 140 with poor response. The radiomics model demonstrated that the AUC was higher in the combined model than in the intratumoral and peritumoral models (training group: 0.949, 0.943, and 0.846, respectively; validation group: 0.815, 0.778, and 0.701, respectively). Age, Borrmann classification, and Lauren classification were used to construct the clinical model. Among the radiomics–clinical models, the combined-clinical model showed the highest AUC (training group: 0.960; validation group: 0.843), which significantly improved prediction efficiency.

**Conclusion:**

The peritumoral model provided additional value in the evaluation of pathological response after neoadjuvant chemotherapy against advanced gastric cancer, and the combined-clinical model showed the highest predictive efficiency.

**Critical relevance statement:**

Intratumoral and peritumoral radiomics can noninvasively predict the pathological response against advanced gastric cancer after neoadjuvant chemotherapy to guide early treatment decision and provide individual treatment for patients.

**Key points:**

1. Radiomics can predict pathological responses after neoadjuvant chemotherapy against advanced gastric cancer.

2. Peritumoral radiomics has additional predictive value.

3. Radiomics–clinical models can guide early treatment decisions and improve patient prognosis.

**Graphical Abstract:**

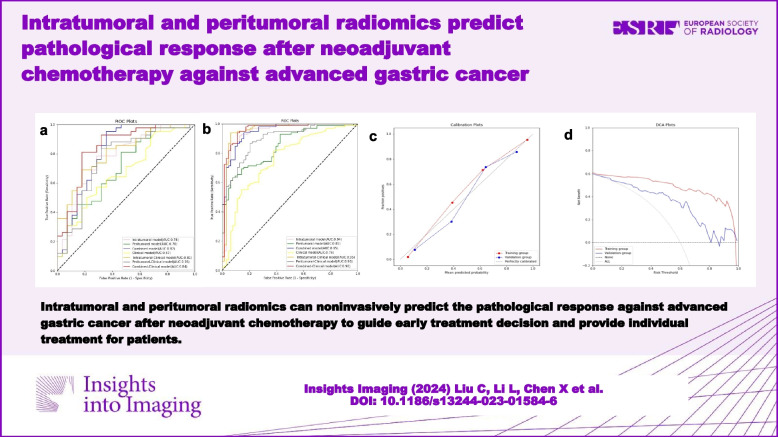

**Supplementary Information:**

The online version contains supplementary material available at 10.1186/s13244-023-01584-6.

## Background

Gastric cancer is the fifth most common cancer and third most common cause of cancer-related deaths worldwide [1]. Although the incidence of gastric cancer is constantly decreasing, in 2020, 1,089,103 people were diagnosed with gastric cancer worldwide, and China accounted for about 44% of the cases [2]. More than 80% of the cases were already in an advanced stage at the time of treatment, and prognosis was poor [3]. Neoadjuvant chemotherapy (NAC) can reduce tumor staging, increase R0 resection rate, reduce recurrence and metastasis, and thus improve prognosis [4], hence, NAC is now a recommended treatment option for patients with advanced gastric cancer [5]. Both the National Comprehensive Cancer Network (NCCN) [6] and the Chinese Society of Clinical Oncology (CSCO) [3] recommend NAC for patients with advanced gastric cancer. However, not all patients benefit from NAC, and at least 20% of patients fail to achieve pathological remission after NAC [7], which exposes patients to potential side effects without any benefit, resulting in delayed surgery, tumor progression, and poor prognosis. Therefore, early determination of the pathological response to NAC is of great significance to reduce chemotherapy toxicity and guide individualized treatment strategies.

Radiomics analysis may be used to characterize tumor heterogeneity based on high-throughput image quantitative feature extraction, with good application potential in disease diagnosis, tumor staging, and prognosis prediction [8–12]. Previous studies have shown that intratumoral radiomics models can effectively predict the pathological response of advanced gastric cancer after NAC [13–15]. In addition, certain features of the peritumoral region may be exploited to predict the tumor pathological response. Braman et al. [16] showed that peritumoral radiomics is of great significance in predicting the pathological response after NAC in different breast cancer subtypes. Hu et al. [17] established a model based on peritumoral radiomics features of esophagogastric junction cancer, which can effectively predict the pathological remission of tumors after NAC. Sun [18] and Khorrami et al. [19] confirmed the predictive ability of the peritumoral region.

Previous radiomics studies on the pathological response of advanced gastric cancer after NAC have mainly focused on the intratumoral region [13–15], and no studies on the peritumoral region have been reported. Our hypothesis is that peritumoral radiomics may contribute to the prediction of pathological responses after NAC in advanced gastric cancer. Therefore, this study aimed to explore the predictive value of peritumoral radiomics for pathological response after NAC in advanced gastric cancer and to establish intratumoral, peritumoral, and combined models based on enhanced CT. In addition, a clinical model was established and combined with a radiomics model to further improve the predictive efficacy of pathological responses after NAC in advanced gastric cancer.

## Methods

The study protocol was approved by the Medical Ethics Committee of Zhengzhou University and the need for informed consent was waived.

### Patient selection

Data from 385 patients with advanced gastric cancer who underwent NAC at the First Affiliated Hospital of Zhengzhou University between July 2014 and February 2022 were retrospectively collected. The following inclusion criteria were applied: (1) gastric cancer confirmed with pathological biopsy before NAC and clinical stage was cT_2-4a_N_x_M_0_; (2) abdominal enhanced CT examination performed within one week before NAC; (3) no distant metastasis; (4) gastrectomy performed after NAC was completed according to the established protocol; and (5) complete clinical, pathological, and CT data. The following exclusion criteria were applied to the 385 patients: (1) history of previous abdominal surgery (*n* = 24); (2) presence of other malignant tumors (*n* = 17); (3) poor gastric filling or tumor could not be identified at CT (*n* = 75); (4) poor computed tomography (CT) image quality with many artifacts (*n* = 16); and (5) other antitumor therapy before NAC (*n* = 22). Thus, a total of 231 patients remained for further analysis.

### NAC regimens and pathological response assessment

Enrolled patients were treated according to the prescribed treatment plan, which included oxaliplatin + S-1 (SOX), 5-fluorouracil + leucovorin + oxaliplatin + docetaxel (FLOT), and capecitabine + oxaliplatin (XELOX). Postoperative pathological specimens were graded according to the Becker grading system [20], as follows: grade 1a, no residual tumor cells; grade 1b, < 10% residual tumor cells; grade 2, 10–50% residual tumor cells; grade 3, > 50% residual tumor cells. In this study, patients with grades 1a and 1b were classified as having good response (GR). Patients with grades 2 and 3 were classified as having poor response (PR).

### Clinical data

The clinical data collected in this study included sex, age, tumor location, Borrmann classification, clinical T stage (cT), clinical N stage (cN), tumor thickness, histopathology, differentiation degree, Lauren classification, and levels of carcinoembryonic antigen (CEA), carbohydrate antigen 125 (CA125), and carbohydrate antigen 199 (CA199). The tumor location was classified as fundus, body, or antrum (tumors of the gastroesophageal junction were not included), and the extent of gastric wall involvement was ≥ 2/3. Borrmann type I: The tumor was lumpy and protruded into the lumen. Borrmann type II: The center of the tumor is ulcerated and the boundary is clear. Borrmann Type III: There are also ulcers formed with incomplete edges and blurred boundaries. Borrmann type IV: Diffuse thickening and stiffness of the gastric wall. cT and cN were evaluated according to the 8th edition of the American Joint Committee on Cancer (AJCC) criteria [21]. To measure tumor thickness, the largest layer of the tumor was selected and measured at the thickest part. Histopathology included adenocarcinoma and non-adenocarcinoma; non-adenocarcinoma included signet ring cell carcinoma and mucinous adenocarcinoma. Lauren classification includes intestinal type, mixed type, and diffuse type. CEA, CA125, and CA199 were changed into dichotomous data (normal and elevated) according to the respective reference values (CEA ≤ 3.4 μg/L, CA125 ≤ 35 μg/L, and CA199 ≤ 27 μg/L).

### CT image acquisition

All patients were scanned using a 64-slice CT scanner (Discovery CT 750 HD; GE Healthcare, Waukesha, WI, USA, or Siemens Sensation 64 CT; Siemens Healthcare, Forchheim, Germany). Patients were instructed to fast for at least 6 h before scanning and to consume 500–1000 ml of water orally to dilate the stomach cavity before the examination. Patients were positioned supine and scanning ranged from the top of the diaphragm to the upper margin of the pubic symphysis for both plain and contrast-enhanced scans. First, a plain CT scan was performed. During enhancement examination, a high-pressure power injector (Urich REF XD 2060-Touch, Ulrich Medical) was used to inject the contrast agent iohexol (Shanghai Bolaike Xinyi Pharmaceutical Co., Ltd., iodine concentration 370 mg/mL) through the elbow vein at a flow rate of 3.5 mL/s and a dose of 1.5 mL/kg. The arterial phase scan started 25 to 30 s, and the venous phase scan started 60 to 70 s after contrast agent injection, with the following scanning parameters: tube voltage 120 kV, tube current 220–330 mA or automatic milliampere second technology, field of view (FOV) 35–50 cm, matrix 512 × 512, rotation time 0.5–0.8 s, pitch 1.375 or 1.1, and reconstruction layer thickness 2 mm.

### ROI (region of interest) segmentation

**Fig. 1:**
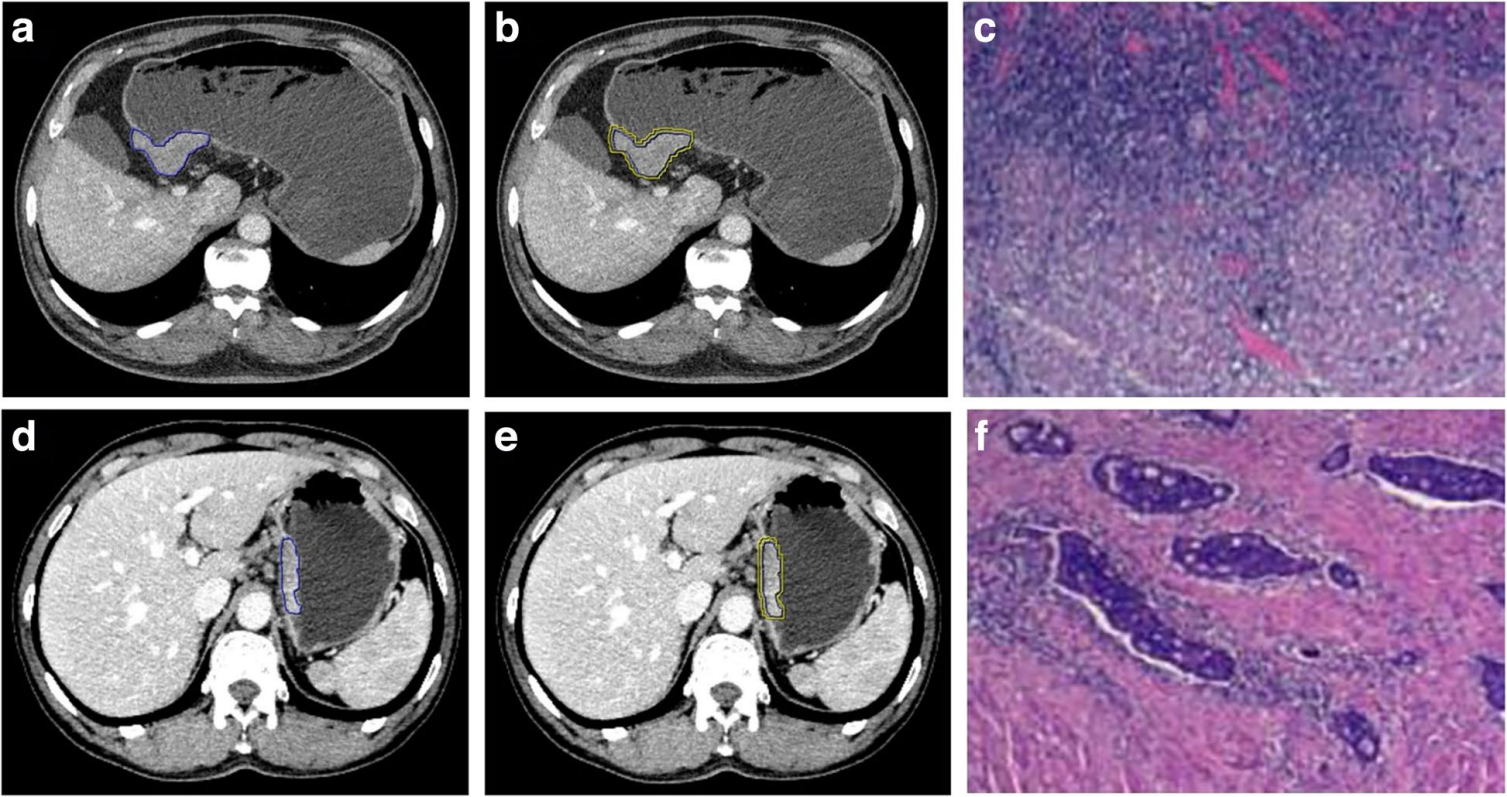
ROI segmentation diagram. **a**–**c** A 61-year-old male patient with moderately differentiated advanced gastric adenocarcinoma. **a** The tumor was located in the gastric antrum, and the intratumoral ROI was delineated along the tumor edge. **b** The boundary was equidistant outward by 2 mm to form the peritumoral ROI. **c** Postoperative pathological picture: Massive chronic inflammatory cell infiltration, no residual tumor cells, grade 1a (HE × 200). **d**–**f** A 59-year-old male patient with moderately differentiated advanced gastric adenocarcinoma. **d** The tumor was located in the cardia, and the intratumoral ROI was delineated along the tumor edge. **e** Boundary was equidistant outward by 2 mm to form the peritumoral ROI. **f** Postoperative pathological picture: Proliferation of interstitial fibrous tissue and a small amount of tumor cell degeneration, grade 3 (HE × 200)

Thin-layer images of patients in the venous phase were retrieved from the picture archiving and communication system (PACS) and stored in Digital Imaging and Communications in Medicine (DICOM) format. Images were uploaded to the Dr. Wise Multimodal Research Platform (https://keyan.deepwise.com). Region of interest (ROI) for gastric cancer was delineated layer-by-layer on the axial image by a radiologist with 3 years of experience (C.C.L.), and the delineation process was supervised by a radiologist with 5 years of experience (L.M.L.). Each layer of gastric cancer lesions was sketched along the edge of the lesion, and the obvious necrotic area was avoided as far as possible when delineating. After the outline of the lesion ROI was completed, a peripheral ring of 2 mm was automatically created using the automatic outward expansion function of the software (Fig. 1). To ensure reliability and repeatability of the radiomics features, ROIs from 30 randomly selected patients (training group: 20, validation group: 10) were delineated again one month later by the same radiologists (C.C.L. and L.M.L.).

### Radiomics feature extraction

After all ROIs were delineated, intratumoral, peritumoral, and combined radiomics features were automatically extracted using appropriate software. Image preprocessing methods included the original image, wavelet transform, Laplacian of gaussian transform (LoG), local binary pattern applied in 2D (LBP2D), local binary pattern applied in 3D (LBP3D), square, square root, logarithm, exponential, and gradient. Radiomics features included first-order features, shape features, gray-level co-occurrence matrix (GLCM), gray-level size zone matrix (GLSZM), gray-level run length matrix (GLRLM), gray-level dependence matrix (GLDM), and neighborhood grayscale difference matrix (NGTDM). These features are commonly used classic features [22].

### Radiomics feature selection

Feature selection was performed according to the following three steps:

1. The consistency of the extracted radiomics features was tested, and radiomics features with intraclass correlation coefficient (ICC) ≥ 0.80 were screened.
2. The screened features were normalized using the following formula: *z* = (*x* − mean)/std, where std stands for standard deviation. Then, correlation analysis of the features was performed to alleviate redundancy between features, and the threshold of the correlation analysis was 0.55.
3. Lasso was used to further reduce dimensionality and screen out features with larger absolute coefficients.

### Model establishment

In the training group, influencing indicators related to pathological responses were screened using univariate analysis, and independent risk factors related to pathological responses were screened using multivariate logistic regression analysis. A clinical model was established based on independent risk factors, and the predicted probability of the model output was considered as the clinical score. In the validation group, the same clinical influencing indicators were used to establish and validate the clinical model.

Based on the selected radiomics features, SVM classifiers [23] were used to establish intratumoral, peritumoral, and combined models, and the prediction probability output of the model was considered as the radiomics score (RS) for each model.

The predicted probabilities of the output of the radiomics and clinical models were combined, and a logistic regression analysis was performed to establish the radiomics–clinical model. The radiomics–clinical model included intratumoral-clinical, peritumoral-clinical, and combined-clinical models.

### Statistical analysis

The Shapiro–Wilk test was used to analyze the normality of the measurement data using the SPSS 25.0 statistical analysis software. Non-normally distributed data were expressed as M (Q1, Q3). The Mann–Whitney *U* test was used to compare the measurement data between the training and validation groups, and the chi-square test or Fisher’s exact test was used to compare the count data. Statistical significance was set at *p* < 0.05.

The R software was used to draw receiver operating characteristic (ROC) curves for each model, and the area under curve (AUC), accuracy, sensitivity, specificity, positive predictive value (PPV), and negative predictive value (NPV) were used to evaluate the prediction efficiency of the model. Hosmer–Lemeshow test were used to evaluate the correction ability of the model. In the Hosmer–Lemeshow test, *p* > 0.05 indicated no significant difference between the predicted and real value [22]. Calibration curves and decision curves were used to evaluate the optimal model.

The Delong test was used to compare the AUC of different models using MedCalc 19.0.2, and statistical significance was set at *p* < 0.05.

## Results

### Baseline characteristics

A total of 231 patients with advanced gastric cancer were randomly divided into a training group (161 patients) and a validation group (70 cases) at a ratio of 7:3. Of these, 182 were male and 49 were female. Patient age ranged from 23 to 76 years, with a median of 61 years. Out of 161 cases in the training group, 63 cases had GR, accounting for 39.13%, and 98 cases had PR, accounting for 60.87%. A total of 70 cases comprised the validation group, including 28 cases with GR (40.00%) and 42 cases with PR (60.00%).

### Clinical model

In the training group, univariate analysis showed that age, Borrmann classification, and Lauren classification were significantly different between patients with GR and those with PR (Table 1). Multivariate logistic regression analysis showed that age, Borrmann classification, and Lauren classification were independent risk factors for predicting pathological response after NAC in advanced gastric cancer (Table S1). Clinical models were established based on the age, Borrmann classification, and Lauren classification.

**Table 1:** Comparison of clinical, pathological and imaging parameters between training and validation groups

	Training group		*p* value	Validation group		*p* value
	GR (63)	PR (98)		GR (28)	PR (42)	
Gender			0.471^a^			0.062^a^
Male	53	78		17	34	
Female	10	20		11	8	
Age (years)	58.00 (52.00, 66.00)	62.00 (56.00, 69.00)	0.031^b^	61.00 (57.00, 65.75)	63.00 (58.00, 67.25)	0.387^b^
Tumor location			0.113^c^			0.079^c^
Fundus	42	60		18	26	
Body	10	27		2	11	
Antrum	11	9		7	4	
≥ 2/3	0	2		1	1	
Borrmann			0.031^c^			0.907^c^
I	3	5		2	4	
II	50	60		22	29	
III	10	33		4	8	
IV	0	0		0	1	
Clinical T stage			0.688^a^			0.492^c^
2	6	7		4	3	
3	43	64		16	29	
≥ 4a	14	27		8	10	
Clinical N stage			0.437^a^			0.411^a^
N0	27	36		8	16	
N +	36	62		20	26	
Thickness (mm)	13.82 (9.88, 16.16)	13.02 (10.08, 16.47)	0.609^b^	13.78 (11.03, 16.33)	13.16 (10.48, 16.87)	0.971^b^
Histopathology			0.091^a^			0.552^a^
Adenocarcinoma	55	75		23	32	
Non-adenocarcinoma	8	23		5	10	
Differentiation			0.102^c^			0.146^a^
Well	1	0		0	0	
Moderate	38	48		12	11	
Poor	24	50		16	31	
Lauren			0.001^a^			0.117^a^
Intestinal type	44	41		17	15	
Mixed type	6	27		6	16	
Diffuse type	13	30		5	11	
CEA			0.069^a^			0.188^a^
Normal	49	63		18	33	
Elevated	14	35		10	9	
CA125			0.942			0.383
Normal	57	89		25	40	
Elevated	6	9		3	2	
CA199			0.657			0.714
Normal	51	82		23	33	
Elevated	12	16		5	9	

*GR* Good response, *PR* Poor response, *CEA* Carcinoembryonic antigen, *CA125* Carbohydrate antigen 125, *CA199* Carbohydrate antigen 199

^a^*χ*^2^ value

^b^*Z* value

^c^Fisher’s exact probability method

### Radiomics model

Based on the original and filtered images, 2107 radiomics features were extracted from intratumoral and peritumoral images, and 4214 radiomics features were extracted from the combined intratumoral and peritumoral images. Among the intratumoral, peritumoral, and combined radiomics features, 1618, 1778, and 3288 features, respectively, were screened (ICC ≥ 0.80). After three feature selection steps, 36 radiomics features were included in the intratumoral model, 13 in the peritumoral model, and 29 in the combined model. The specific radiomics features are listed in Table S2. Based on the above features, an SVM classifier was used to establish intratumoral, peritumoral, and combined models.

### Evaluation of model performance

The prediction probabilities of the intratumoral, peritumoral, and combined models were combined with the prediction probabilities of the clinical model, and the new prediction probabilities were obtained by logistic regression analysis to establish the intratumoral-clinical model, peritumoral-clinical model, and combined-clinical model.

**Fig. 2:**
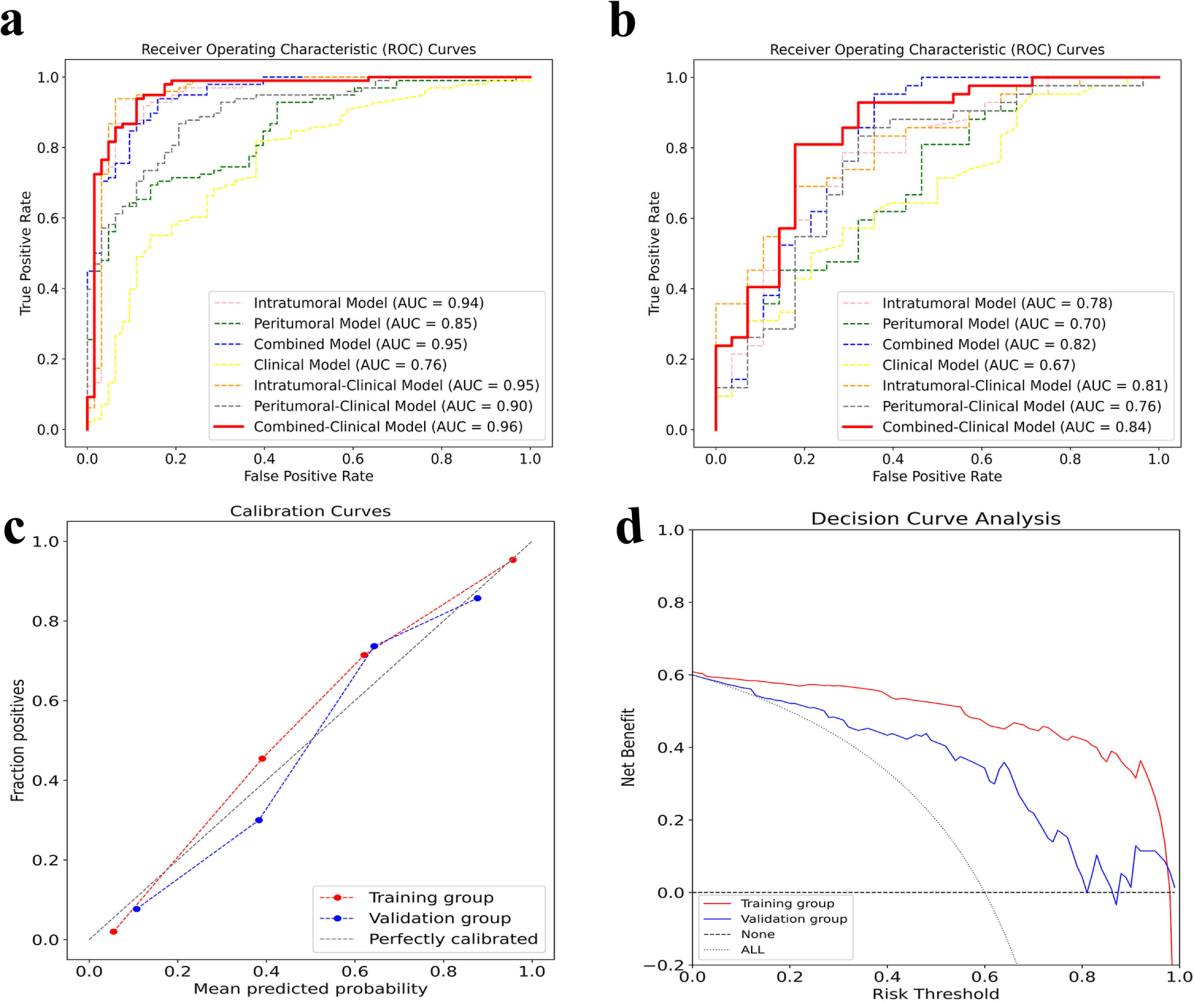
The performance of different models. **a** ROC curves of each model in the training group. **b** ROC curves of each model in the validation group. **c** Calibration curve of optimal model. **d** Decision curve of optimal model

Seven models were established for this study. The AUC, accuracy, sensitivity, specificity, PPV, and NPV of each model for predicting pathological responses after NAC in advanced gastric cancer are shown in Table 2. The ROC curves for each model are shown in Fig. 2. The results of the Delong test for pairwise comparisons of all models in the training and validation groups are shown in Table 3. By observing the predictive efficacy index of the model, it can be seen that in the training and validation group, the predictive efficacy of the combined model is better than that of the single intratumoral or peritumoral model. After combining with the clinical model, the predictive efficacy of the radiomics–clinical model has an increasing trend, among which the combined-clinical model has the best predictive performance. The AUC reached 0.960 in the training group and 0.843 in the validation group. The calibration curve of the combined-clinical model showed good fit, and the decision curve showed the clinical benefit of the model (Fig. 2). The Hosmer–Lemeshow test (Table S3) for each model showed good fit.

**Table 2:** Efficacy of different models in predicting pathological responses after NAC in advanced gastric cancer in the training and validation groups

Model	Intratumoral model	Peritumoral model	Combined model	Clinical model	Intratumoral-clinical model	Peritumoral-clinical model	Combined-clinical model
Training group							
AUC	0.943	0.846	0.949	0.756	0.955	0.898	0.960
95% CI	0.896–0.974	0.781–0.898	0.903–0.978	0.682–0.820	0.910–0.981	0.840–0.940	0.917–0.984
*p* value	< 0.0001	< 0.0001	< 0.0001	< 0.0001	< 0.0001	< 0.0001	< 0.0001
ACC (%)	89.44	74.53	90.06	73.91	93.79	83.85	91.93
SEN(%)	86.73	63.27	93.88	81.63	93.88	86.73	93.88
SPE(%)	93.65	92.06	84.13	61.90	93.65	79.37	88.89
PPV(%)	95.51	92.54	90.20	76.92	95.83	86.73	92.93
NPV(%)	81.94	61.70	89.83	68.42	90.77	79.37	90.32
Validation group							
AUC	0.778	0.701	0.815	0.672	0.808	0.759	0.843
95% CI	0.663–0.869	0.579–0.804	0.705–0.898	0.549–0.779	0.696–0.892	0.641–0.853	0.736–0.919
*p* value	< 0.0001	0.002	< 0.0001	0.0093	< 0.0001	< 0.0001	< 0.0001
ACC (%)	75.71	70.00	82.86	62.86	74.29	77.14	81.43
SEN(%)	78.57	80.95	95.24	57.14	69.05	83.33	80.95
SPE(%)	71.43	53.57	64.29	71.43	82.14	67.86	82.14
PPV(%)	80.49	72.34	80.00	75.00	85.29	79.55	87.18
NPV(%)	68.97	65.22	90.00	52.63	63.89	73.08	74.19

*AUC* Area under the curve, *CI* Confidence interval, *ACC* Accuracy, *SEN* Sensitivity, *SPE* Specificity, *PPV* Positive predictive value, *NPV* Negative predictive value

**Table 3:** *p* value of Delong test for any two models in the training and validation groups

	Intratumoral model	Peritumoral model	Combined model	Clinical model	Intratumoral-clinical model	Peritumoral-clinical model
Training group						
Peritumoral model	0.002					
Combined model	0.704	0.0001				
Clinical model	< 0.0001	0.082	< 0.0001			
Intratumoral-clinical model	0.430	0.001	0.794	< 0.0001		
Peritumoral-clinical model	0.059	0.010	0.020	0.0001	0.016	
Combined-clinical model	0.299	0.0001	0.315	< 0.0001	0.737	0.003
Validation group						
Peritumoral model	0.259					
Combined model	0.423	0.060				
Clinical model	0.245	0.747	0.130			
Intratumoral-clinical model	0.384	0.087	0.891	0.044		
Peritumoral-clinical model	0.788	0.195	0.432	0.137	0.366	
Combined-clinical model	0.167	0.015	0.366	0.022	0.401	0.126

## Discussion

The onset of gastric cancer is insidious and early symptoms lack specificity. Most patients are already at an advanced stage when they visit the hospital [24]. NAC provides the possibility for radical resection in these patients [25–28]. However, some patients still suffer from unnecessary chemotherapy toxicity and the survival rate after NAC is not improved [10, 29]. Currently, tumor regression grading (TRG) of postoperative pathological specimens is a reliable indicator for evaluating prognosis of gastric cancer [30]; however, TRG can only be determined after surgery and cannot be used as the basis for guiding treatment. Therefore, it is very important to accurately predict the pathological response to NAC before treatment to enhance individualized treatment and clinical decision-making in patients with advanced gastric cancer. This study was based on pre-NAC enhanced CT venous phase images, establishment of intratumoral, peritumoral, and combined models, and combination of the clinical model with the radiomics model. The results showed that among the radiomics models, the combined model was more effective than the intratumoral and peritumoral models in predicting pathological responses after NAC. The peritumoral model provided additional value in evaluating the pathological response, and the combined-clinical model had the highest predictive efficiency.

Previous studies have demonstrated the value of intratumoral models in predicting pathological responses after NAC in advanced gastric cancer. Most AUCs ranged from 0.621 to 0.770 [7, 13, 31, 32]. Our results showed that the AUC of the intratumoral model in the training and validation groups were 0.943 and 0.778, respectively. The AUC of the validation group was not significantly different from that of previous studies, which further confirmed the value of tumor heterogeneity reflected by intratumoral radiomics features in predicting pathological responses after NAC. Notably, in our study, 3D delineation was used for image analysis. Compared with previous 2D delineation, this method can provide more comprehensive tumor information, extract more stable and accurate features, and monitor more detailed tumor heterogeneity information. This may be the reason why the AUC of this study is slightly higher than that of previous studies [7, 13, 31, 32]. The predictive performance of the radiomics model is closely related to the appropriate classifier. Considering the complexity and nonlinearity of the relationship between radiomics features and pathological responses, the SVM classifier was used to construct radiomics models in this study, which effectively and robustly solved the nonlinear problem.

The tumor immune microenvironment, which is important for tumor progression, metastasis, and treatment effect, has attracted increasing attention [33–36]. A previous study [37] showed that peritumoral lymphocyte infiltration in gastric cancer was significantly correlated with prognosis and chemotherapy response. When infiltration of CD3 and CD8 cells into the tumor microenvironment is lower, the prognosis of patients is good, but the chemotherapy response is poor [37]. Additionally, peritumoral neutrophil infiltration can also promote tumor development, resulting in a low response to chemotherapy [38]. Liu et al. [39] also reported that infiltration of CD20 + B cells around gastric cancer was may independently predict prognosis of gastric cancer. Patients with high immune cell infiltration have a shorter survival time but are more likely to benefit from chemotherapy. Radiomics analysis can monitor the immune cell microenvironment and reveal the relationship between peritumoral heterogeneity and pathological response, which may predict the efficacy of NAC in breast cancer [16, 40], cervical cancer [18], lung adenocarcinoma [19], and esophagogastric junction cancer [17]. However, peritumoral models to predict pathological responses after NAC in advanced gastric cancer are rarely established. Our results showed that the AUC of the peritumoral model in the training and validation groups were 0.846 and 0.701, respectively. When the intratumoral and peritumoral models were combined, the predictive ability of the model further improved, reaching 0.949 in the training group and 0.815 in the validation group. Thus, the additional value of the peritumoral model in predicting pathological responses after NAC in advanced gastric cancer was confirmed.

Clinical indicators were also included in this study. The results of the training group showed that age, Borrmann classification, and Lauren classification were independent risk factors for poor response after NAC in advanced gastric cancer. The median age was higher for the PR group than for the GR group, which was not consistent with results a previous studies based on the response evaluation criteria for solid tumors (RECIST) [41]. We speculate that this is related to the decline of physical function in the elderly and the resistance to chemotherapy drugs [42]. Furthermore, the RECIST are limited due to the varying degree of gastric filling and low reproducibility of data measurements, which may bias the results [22, 43]. Studies have shown that Borrmann type III and IV gastric cancers have a low survival rate and poor prognosis [44, 45], while TRG after NAC was also significantly correlated with prognosis [20]. In our study, the Borrmann classification showed statistically significant differences between GR and PR groups, which is consistent with results from previous studies. Patients with diffuse-type gastric cancer have a poor prognosis and are prone to relapse [46]. Our study also showed a statistically significant difference in Lauren typing between GR and PR groups. Previous studies have shown that the degree of tumor differentiation, cT, cN, and other clinical indicators were related to pathological response; however, these results remain controversial [13, 47, 48]. This may be due to a lack of large population trials or multicenter studies. In our study, the sample size of the validation group was smaller than that of the training group, so the independent risk factors in the training group were not replicated in the validation group. However, considering the clinical significance of these indicators, we used these indicators to build clinical models.

The limitations of this study are as follows: (1) it is a single-center retrospective study with confounding factors and limited generalization of results, which needs to be further validated in a multicenter prospective cohort; (2) delineation of ROI is subjective, and peritumoral expansion inevitably includes part of the gastric cavity contents, which may bias the results; an automatic or semi-automatic image analysis method should be established to improve accuracy in the future; (3) different NAC regimens may bias the results, and the sample size should be expanded and stratified for analysis; (4) the baseline characteristics of patients, including gender ratio and age range, are slightly different. Although the majority of gastric cancer patients are male, and big data studies show that cancer is getting younger, these differences may limit the study to some extent, and the sample size should be expanded for further study in the future.

## Conclusions

In conclusion, the peritumoral model has additional value in the evaluation of pathological responses after NAC in advanced gastric cancer. The combined model can effectively improve prediction efficiency. The combined-clinical model has the highest prediction efficiency, which can guide early treatment decisions and improve patient prognosis.

### Supplementary Information

**Additional file 1: Table S1.** Multivariate analysis for predicting pathological response after NAC in advanced gastric cancer in training group. **Table S2.** Radiomic features used to build the model. **Table S3.** Hosmer-Lemeshow test results for different models in the training and validation groups.

## Data Availability

The dataset used or analyzed during the current study are available from the corresponding author upon reasonable request.

## Abbreviations

AUC: Area under curve
CA125: Carbohydrate antigen 125
CA199: Carbohydrate antigen 199
CEA: Carcinoembryonic antigen
cN: Clinical N stage
cT: Clinical T stage
CT: Computed tomography
DICOM: Digital Imaging and Communications in Medicine
FLOT: 5-Fluorouracil + leucovorin + oxaliplatin + docetaxel
FOV: Field of view
GLCM: Gray-level co-occurrence matrix
GLDM: Gray-level dependence matrix
GLRLM: Gray-level run length matrix
GLSZM: Gray-level size zone matrix
GR: Good response
ICC: Intraclass correlation coefficient
LBP2D: Local binary pattern applied in 2D
LBP3D: Local binary pattern applied in 3D
LoG: Laplacian of gaussian transform
NAC: Neoadjuvant chemotherapy
NCCN: National Comprehensive Cancer Network
NGTDM: Neighborhood grayscale difference matrix
NPV: Negative predictive value (NPV)
PACS: Picture archiving and communication system
PPV: Positive predictive value
PR: Poor response
RECIST: Response evaluation criteria for solid tumors
ROC: Receiver operating characteristic (ROC)
ROI: Region of interest
RS: Radiomics score
SOX: Oxaliplatin + S-1
SVM: Support vector machine
XELOX: Capecitabine + oxaliplatin
