# Supplementary material for: Intratumoral and peritumoral radiomics predict pathological response after neoadjuvant chemotherapy against advanced gastric cancer
Source: Insights Imaging. 2024 Jan 25;15:23. doi: 10.1186/s13244-023-01584-6 (PMC10811314; doi:10.1186/s13244-023-01584-6)
Supplement: Supplementary file 1 — Additional file 1: Table S1. Multivariate analysis for predicting pathological response after NAC in advanced gastric cancer in training group. Table S2. Radiomic features used to build the model. Table S3. Hosmer-Lemeshow test results for different models in the training and validation groups. [file 13244_2023_1584_MOESM1_ESM.pdf]

Intratumoral and peritumoral radiomics predict pathological response after neoadjuvant chemotherapy against advanced gastric cancer

ELECTRONIC SUPPLEMENTARY MATERIAL

Table S1. Multivariate analysis for predicting pathological response after NAC in advanced gastric cancer in training group.

Characteristic	b value	standard error	Waldχ^2	*P* value	OR（95%CI）
Age	0.054	0.020	7.194	0.007	1.056（1.015～1.098）
Borrmann			6.264	0.044	
Borrmann(1)	-0.436	0.799	0.298	0.585	0.646（0.135～3.093）
Borrmann(2)	0.656	0.860	0.581	0.446	1.927（0.357～10.405）
Lauren			14.275	0.001	
Lauren(1)	1.758	0.518	11.523	0.001	5.798（2.102～15.996）
Lauren(2)	1.072	0.432	6.176	0.013	2.922（1.254～6.808）
constant	-3.242	1.446	5.027	0.025	0.039

CI: confidence interval

Table S2. Radiomic features used to build the model.

Model	Radiomic features
Intratumoral model	log-sigma-5-0-mm-3D_ngtdm_Busyness
	log-sigma-4-0-mm-3D_glcm_Idn
	wavelet-HLH_firstorder_Median
	wavelet-LLH_glszm_ZoneEntropy
	log-sigma-1-0-mm-3D_firstorder_Kurtosis
	lbp-3D-m2_firstorder_RootMeanSquared
	wavelet-LHH_firstorder_Kurtosis
	wavelet-LLH_glszm_SmallAreaEmphasis
	original_shape_Sphericity
	log-sigma-5-0-mm-3D_gldm_DependenceVariance
	square_gldm_HighGrayLevelEmphasis
	square_glszm_LowGrayLevelZoneEmphasis

	wavelet-HLH_firstorder_Skewness
	wavelet-HHH_ngtdm_Strength
	lbp-2D_glszm_SmallAreaEmphasis
	original_glcm_Correlation
	wavelet-HLH_gldm_DependenceVariance
	wavelet-LLH_gldm_DependenceNonUniformityNormalized
	wavelet-LHH_glcm_InverseVariance
	log-sigma-1-0-mm-3D_glszm_SmallAreaEmphasis
	original_glrlm_RunVariance
	wavelet-HLH_firstorder_RootMeanSquared
	log-sigma-4-0-mm-3D_glszm_GrayLevelNonUniformity
	lbp-2D_firstorder_10Percentile
	wavelet-LLL_firstorder_90Percentile
	log-sigma-1-0-mm-3D_gldm_DependenceNonUniformityNormalized
	logarithm_glcm_InverseVariance
	square_ngtdm_Busyness
	exponential_firstorder_Kurtosis
	wavelet-LHH_firstorder_RootMeanSquared
	wavelet-LHL_firstorder_Skewness
	lbp-3D-m1_firstorder_Median
	wavelet-HHH_gldm_SmallDependenceLowGrayLevelEmphasis
	wavelet-HHL_glcm_InverseVariance
	lbp-2D_firstorder_90Percentile
	log-sigma-5-0-mm-3D_glszm_SmallAreaEmphasis
Peritumoral model	wavelet-LHH_firstorder_Kurtosis
	wavelet-LHL_glszm_ZoneEntropy
	lbp-3D-k_firstorder_Maximum
	log-sigma-4-0-mm-3D_glszm_LargeAreaEmphasis
	wavelet-LHH_glcm_InverseVariance
	gradient_ngtdm_Coarseness
	wavelet-LLH_ngtdm_Contrast
	exponential_glszm_SizeZoneNonUniformity
	lbp-2D_glszm_ZoneVariance
	wavelet-LLH_firstorder_Skewness
	log-sigma-2-0-mm-3D_firstorder_Median
	wavelet-HHH_firstorder_Skewness
	wavelet-HLL_firstorder_Median
Combined model	log-sigma-5-0-mm-3D_ngtdm_Busyness_1
	wavelet-LHH_firstorder_Kurtosis
	lbp-3D-k_firstorder_Maximum
	wavelet-LLH_glszm_ZoneEntropy_1
	log-sigma-4-0-mm-3D_glszm_LargeAreaEmphasis
	lbp-3D-m2_firstorder_RootMeanSquared_1
	original_shape_Sphericity_1

square_gldm_HighGrayLevelEmphasis_1
wavelet-HLH_firstorder_Skewness_1
lbp-2D_glszm_SmallAreaEmphasis_1
original_glcm_Correlation_1
wavelet-HLH_gldm_DependenceVariance_1
wavelet-LHH_glcm_InverseVariance
wavelet-LLH_gldm_DependenceNonUniformityNormalized_1
wavelet-LLH_ngtdm_Contrast
original_glrlm_RunVariance_1
wavelet-HLH_firstorder_RootMeanSquared_1
log-sigma-1-0-mm-3D_gldm_DependenceNonUniformityNormalized_1
square_ngtdm_Busyness_1
exponential_firstorder_Kurtosis_1
lbp-2D_glszm_ZoneVariance
wavelet-HLL_firstorder_Skewness_1
wavelet-LHH_firstorder_RootMeanSquared_1
wavelet-LLH_firstorder_Skewness
wavelet-LHL_firstorder_Skewness_1
log-sigma-2-0-mm-3D_firstorder_Median
wavelet-HHH_firstorder_Skewness
wavelet-HLL_firstorder_Median
lbp-2D_firstorder_90Percentile_1

Table S3. Hosmer-Lemeshow test results for different models in the training and validation groups.

Model	Training group		Validation group	
	statistics	P value	statistics	P value
Intratumoral model	7.646	0.469	6.583	0.582
Peritumoral model	15.048	0.058	3.857	0.870
Combined model	7.446	0.489	10.667	0.221
Clinical model	4.257	0.833	4.798	0.779
Intratumoral- Clinical model	3.542	0.896	5.502	0.703
Peritumoral- Clinical model	13.379	0.099	8.557	0.381
Combined- Clinical model	6.356	0.607	9.909	0.271
